# The role of lipid metabolism and peroxisome proliferator activation in mediating pro-cancer phenotypes of poly- and perfluoroalkyl substances in testicular cancer

**DOI:** 10.1016/j.etap.2025.104866

**Published:** 2025-11-12

**Authors:** Raya I. Boyd, Doha Shokry, Brayden C. Rennels, Younan Adam, Christine Powell, Samantha Johnson, Michael J. Spinella, Ratnakar Singh

**Affiliations:** Department of Comparative Biosciences, Carl R. Woese Institute for Genomic Biology, Cancer Center of Illinois, University of Illinois Urbana-Champaign, Urbana, IL 61801, United States

**Keywords:** PFAS, Testicular cancer, PPAR, Lipid metabolism, HQ115

## Abstract

Poly- and perfluoroalkyl substances (PFAS) are of human health concern as epidemiological studies show significant associations with testicular germ cell tumors (TGCTs). Here, the effects of perfluorooctanesulfonic acid (PFOS), lithium bis(trifluoromethylsulfonyl)imide (HQ-115), and hexafluoropropylene oxide dimer acid (GenX) on TGCT cells were investigated. Concentrations (10 nM to 1 μM) modelled PFAS doses in relevant human exposure ranges and acute and short-term timepoints (18 h and 4 days) captured proximal mechanisms of action. Metabolomic studies revealed that HQ-115 altered metabolites associated with steroid biosynthesis and lipid metabolism. Peroxisome-proliferator activation receptor (PPAR) target gene expression was altered upon HQ-115 and GenX exposure. Lastly, PFAS exposure altered the activity of PPARs, in TGCT cells, with the most prominent effects being antagonist activity toward PPARγ. These data support that PFAS may act as fatty acid mimics to modulate fatty acid metabolic and steroidogenic endocrine outcome leading to pro-cancer phenotypes in TGCTs.

## 1. Introduction

Poly- and perfluoroalkyl substances (PFAS) are a class of industrial and commercial chemicals made of fluorocarbon bonds. These chemicals have been in production since the 1940s due to their durability and water- and oil-resistant properties (Paul et al., 2009), and the Environmental Protection Agency estimates that more than 13,000 total PFAS exist (Toxic Substances, 2023). While some PFAS types have been voluntarily phased out of use in the United States, many are still present in the environment due to their long half-lives. In addition, perfluorochemicals are resistant to biodegradation, resulting in long residence times in the environment and body, with a serum half-life for some PFAS of up to two to five years (Sunderland et al., 2019). Several communities near chemical plants manufacturing PFAS have documented serum levels over 50-fold higher than the general population due to contaminated drinking water (Sunderland et al., 2019). Human epidemiological studies have found that exposure to two legacy PFAS, perfluorooctanoic acid (PFOA) and perfluorooctanesulfonic acid (PFOS), is associated with various negative health outcomes, including elevated cholesterol, liver enzyme levels, thyroid disorders, pregnancy-related hypertension, preeclampsia, and cancer (Steenland et al., 2020; Boyd et al., 2022). PFAS exposure has been associated with several male reproductive health issues, such as impaired semen quality, altered reproductive hormone levels, and testicular cancer (Steenland et al., 2020; Boyd et al., 2022).

Testicular germ cell tumors (TGCTs) are a type of testicular cancer primarily affecting 15- to 45-year-old men (Bray et al., 2018). TGCTs are thought to arise *in utero* due to altered differentiation of primordial germ cells into germ cell neoplasia *in situ* (GCNIS) cells. The uncontrolled proliferation of these cells is believed to be triggered by the pubertal testosterone surge (Lobo et al., 2019; Singh et al., 2021). The incidence rate of TGCTs is increasing, with the highest incidence in people of Northern European descent and lowest in people of African descent (Bray et al., 2018). TGCTs, are divided into seminomas and non-seminomas. Seminomas comprise approximately 55 % of all GCNIS-derived tumors, are generally homogenous, and are more common in men aged between 35 and 39 (Lobo et al., 2019; Singh et al., 2021). Non-seminomas can be further divided into subtypes based on their histology. These subtypes are embryonal carcinoma, teratoma, yolk-sac tumor, and choriocarcinoma. While several epidemiological studies have shown a significantly increased risk of testicular cancer with PFAS exposure, there are few mechanistic studies (Steenland et al., 2020; Purdue et al., 2023; Bartell and Vieira, 2021).

We have previously shown that exposure to perfluorooctanesulfonic acid (PFOS), a legacy PFAS, and lithium bis(trifluoromethylsulfonyl) imide (HQ-115), a clean energy PFAS derived from biproducts of lithium batteries, promote TGCT growth in mice and that PFOS alters TGCT metabolomic profiles in ways that alter lipid metabolism pathways, such as fatty acid metabolism and steroid biosynthesis (Boyd et al., 2024; Savvidou et al., 2024). We have also shown that exposure to hexafluoropropylene oxide dimer acid (GenX) and PFOS alters the transcription of lipid metabolism signatures based on RNAseq (Boyd et al., 2024). This is congruent with the theory that PFOS and other PFAS mimic fatty acids and act as ligands for the peroxisome-proliferator activation receptors (PPARs) (Vanden Heuvel et al., 2006). Several rodent studies have shown that steroid biosynthesis, lipid regulation, and PPAR activation are affected by PFAS exposure (Naile et al., 2012; Tian et al., 2019).

PFAS accumulate in the testis of rodents and can cause testis damage and impaired Leydig cell function and Leydig cell tumors (Biegel et al., 1995). PFAS exposure is known to activate liver PPARα to increase expression of CYP19A1 to alter estrogen and testosterone levels to effects Sertoli and Leydig cell function (Klaunig et al., 2012). PFAS may also directly affect Leydig cells, leading to deceased secretion of testosterone (Biegel et al., 1995).

The aim of this study was to further analyze the role of lipid metabolism in mediating pro-cancer phenotypes secondary to PFOS, HQ-115, and GenX exposure at doses relevant to human exposure. We first investigated the effects of PFAS on migration and metabolite production. PFAS exposure did not alter migration of TGCT cells. Metabolomic studies revealed that HQ-115, like PFOS, altered metabolites associated with steroid biosynthesis in TGCT cells, consistent with PFAS acting as endocrine-disrupting chemicals. Further, HQ-115 and GenX, but not PFOS, altered the expression of PPAR target genes in TGCT cells. Lastly, PFAS demonstrated partial-agonist and antagonist activity toward PPARs, especially antagonist activity toward PPARγ in TGCT cells. Our data suggests that PFAS exposure at relevant human exposures may mediate pro-cancer phenotypes through acute effects on lipid metabolism by altering the activity of PPARs and expression of PPAR target genes.

## 2. Materials and methods

### 2.1. PFAS Chemicals

All chemicals were purchased from Sigma. PFOS (CAS# 1763–21–1), GenX (CAS# 13252–13–6) and HQ-115 (CAS# 90076–65–6) were dissolved in DMSO. The design was to study PFAS doses in relevant human exposure ranges (general population, contaminated communities, and occupational) according to the Agency for Toxic Substances and Disease Registry. PFOA human serum levels (1.5 μg/L or 3.5 nM, 3 μg/L or 7 nM, 899 μg/L or 2.1 μM). PFOS (4 μg/L or 8 nM, 10 μg/L or 20 nM, 941 μg/L or 1.9 μM). There is no information of relevant doses in human serum for HQ115 and GenX hence PFOS and PFOA doses were chosen as these are typically the most abundant PFAS found in human serum.

### 2.2. Cell Culture

The human embryonal carcinoma cell line 2102EP was purchased from ATCC and authenticated by karyotyping and short tandem repeat profiling. The 2102EP-C1 cell line is an isogenic-derived cisplatin-resistant clone of 2102EP and described previously (Singh et al., 2019). Cells were maintained in Dulbecco’s Modified Eagle Medium with 10 % fetal bovine serum (GeminiBio, Sacramento, CA, USA), 1 % L-glutamine (Corning, Corning, NY, USA), and 1 % antibiotic/antimycotic (Corning, Corning, NY, USA). Cells were frozen within 1 month of purchase and used within 2 months of resuscitation (passage number 10 or lower). Cells were checked for rounded cobblestone morphology of undifferentiated embryonal carcinoma TGCT cells.

### 2.3. Migration assay

The 2102EP cells were plated with silicon inserts (Ibidi, Fitchburg, WI, USA) to provide uniform wounds. Once confluence occurred, inserts were removed, cells were treated with indicated doses of PFOS (Sigma, St. Louis, MO, USA), HQ-115 (Sigma, St. Louis, MO, USA), or GenX (Chem Scene, Monmouth Junction, NJ, USA), and images were obtained every four hours in the indicated media. Images were taken using an Olympus IX72 microscope (Olympus Life Science Solutions, Tokyo, Japan) using cellSens Standard 2.1 (Olympus Life Science Solutions, Tokyo, Japan). Migration was determined using Image J software v1.54 using the Wound healing size tool (Suarez-Arnedo et al., 2020). The parameters of variance window radius, threshold value, and percentage of saturated pixels were set to 20, 40, and 0.400, respectively. Data are reported as the percentage of the area left compared to the control image. Experiments were performed in biological triplicate and repeated at least once.

### 2.4. GC-MS Metabolite Profiling

Cells were treated with indicated doses of HQ-115 for 4 days. Samples were collected in 800 μL of a 3:2:3 (*v*/*v*/*v*) mixture of acetonitrile/water/isopropanol in biological triplicate. Metabolite profiling was conducted by the Carver Metabolomics Core of the University of Illinois Urbana-Champaign Roy J. Carver Biotechnology Center, as previously described (Southey et al., 2021). Each sample was analyzed using a gas-chromatography-mass spectrometry (GC-MS) system consisting of an Agilent 7890 gas chromatograph (Agilent Technologies, Santa Clara, CA, USA), Agilent 5975 MDS, and MP 7683B autosampler. GC was performed on a ZB-5MS (60 ×0.32 mm ID and 0.25 μm film thickness) capillary column (Phenomenex, Torrance, CA, USA). The inlet and MS interface was 250°C, and the ion source temperature was adjusted to 230°C. A 1 μL sample aliquot was injected with a split ratio of 7:1. The helium carrier gas was held at a 2 mL/minute flow rate. Isothermal heating was set at 70°C for 5 min, followed by an oven temperature increase of 5°C/minute to 310°C, then the final 10 min at 310°C. The MS was operated in positive electron ionization (EI) mode at 69.9 eV ionization energy with the scan range of *m/z* 30–800. Peaks were identified using the Automatic Mass Spectral Deconvolution and Identification System (AMDIS) v2.71 (National Institute of Standards and Technology (NIST), Gaithersburg, MD, USA) software and a custom-build MS database derived from in-house chemical standards and the NIST database for annotation confirmation. For this untargeted metabolomics, the limits of detection for each metabolite ranged between low-to-medium microgram per mL levels depending on the target compound structure. Level of quantification was not determined as only relative concentrations were calculated. All data were normalized to the internal standard (hentriacontanoic acid at 10 mg/mL).

MetaboAnalyst 6.0 was used for pairwise comparisons for cluster heatmap, PCA, and pathway analysis. Features with > 50 % missing values were removed. Variables were removed at a threshold of 50 %, and missing values were replaced with 1/5 of the assumed limit of detection (minimum possible value). Data were log-transformed and auto-scaled for heatmaps. For hierarchical clustering, the “hclust” function was used in the “stat” package with distance measured with “Euclidean” and clustering using “ward.D.” Quantitative enrichment and pathway analysis were performed via metabolite set enrichment analysis (MSEA) using metabolites with KEGG-IDs using the log-transformed and auto-scaled data as described previously (Boyd et al., 2024; Southey et al., 2021) (Supplementary Tables S1)

### 2.5. Real-Time PCR

Cells were treated with indicated concentrations of PFOS, HQ-115, GenX, or the vehicle control. After four days, total cellular RNA was isolated using the RNAeasy Mini Kit (Qiagen, Venlo, Netherlands), and complementary DNAs were synthesized using the iScript Select cDNA Synthesis Kit (Bio-Rad, Hercules, CA, USA). Qualitative real-time PCR assays were performed with PowerUp SYBR Green Master Kit iTaq Universal SYBR Green Supermix (Thermo Fisher, Waltham, MA, USA) and QuantStudio 3 Real-time (Thermo Fisher, Waltham, MA, USA) normalized to β-actin as previously described (Boyd et al., 2024). All primers were designed using Primer-BLAST (https://www.ncbi.nlm.nih.gov/tools/primer-blast/) based on the target gene sequence, ensuring optimal Tm, GC content, and amplicon size. Specificity was verified *in silico* and further validated experimentally by real-time PCR melt curve analysis, confirming single, specific amplification without primer-dimer formation to ensure the reliability of quantitative gene expression analysis. Primer sequences are found in Table 1. All RT-PCR analysis were repeated at least once on fresh biological samples with similar results.

### 2.6. PPAR Reporter Assays

2102EP cells were cotransfected with 50 ng of the 3X multimerized PPRE-luciferase reporter vector, 5 ng of the pRL-TK *Renilla* luciferase reporter vector (Promega), and 50 ng of pBABE puro PPARβ (Brun et al., 1996), pSV Sport PPARγ2 (Tontonoz et al., 1994), pCDNA3.1 PPARA (Pan et al., 2021) or pSG5 PPARα. The pSG5 PPARα plasmid was a gift from the Bruce Spiegelman Lab (Addgene plasmid #22751; http://nt2.net/addgene:22751; RRID:Addgene_22751). The identities of the vectors were confirmed by the Sanger Core of the Roy J. Carver Biotechnology Center at the University of Illinois Urbana-Champaign, using the primers shown in Supplementary Table S2. Cells were transfected with Lipofectamine 3000 Transfection Kit (Thermo Fisher) in serum-free O-MEM media (Gibco). The next day, cells were treated with indicated doses of PFAS or known PPAR ligands GW7647 (PPARα, Selleck), GW0742 (PPARβ, Selleck), or Rosiglitazone (PPARγ, Selleck) as positive controls. After 18 h, the Dual-Luciferase Reporter Assay System (Promega) was used to measure receptor activity. Experiments were performed in biological triplicate and repeated at least once on fresh biological samples with similar results.

### 2.7. Statistics

Student’s *t*-tests or ANOVA were performed to compare two or more than two groups, respectively, using GraphPad Prism 10 version 1. Outliers were removed using Grubbs’ test at an alpha level of 0.05 in GraphPad Prism. *p*-values indicative of non-significance (*p* > 0.05) and significance (*p* ≤ 0.05) were determined. Mean and standard error of the mean were used to describe sample variance. Univariate, multivariate and enrichment analysis of all the metabolomics data were analyzed by MetaboAnalyst 6.0, a web-based platform dedicated for comprehensive metabolomics data analysis. The PCA analysis was performed using the prcomp package within MetaboAnalyst. Enrichment analysis uses metabolite set enrichment analysis (MSEA) for human and mammalian species, based on several libraries containing approximately 6300 metabolite sets.

## 3. Results

### 3.1. PFAS Exposure Does Not Increase the Migration Rate of TGCT Cells

**Fig. 1.**
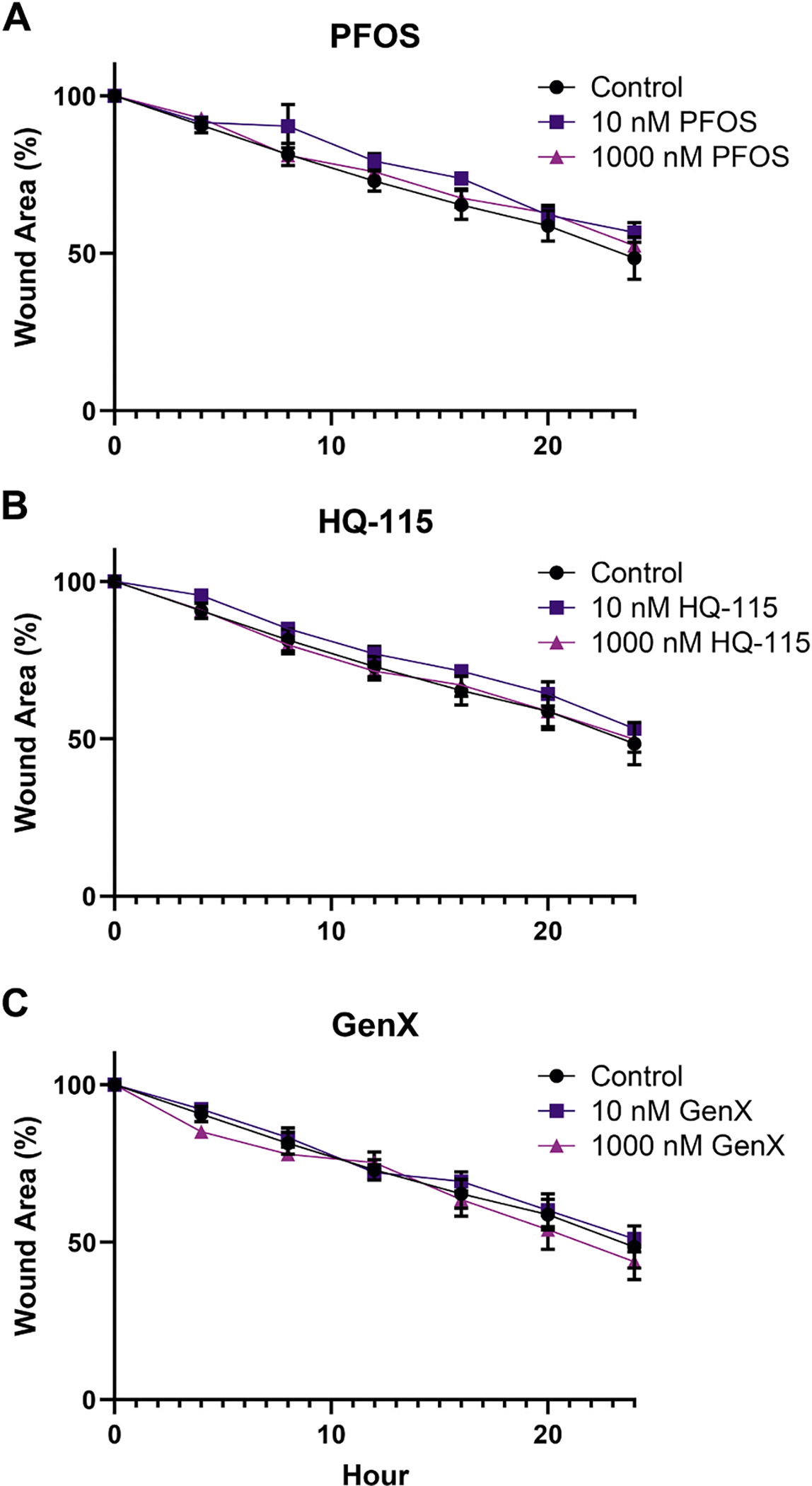
PFAS exposure does not affect migration. 2102EP cells were exposed to vehicle control or the indicated doses of (A) PFOS, (B) HQ-115, or (C) GenX. Unpaired *t*-tests were performed compared to the control for statistical analysis. Data represent mean ± standard error of the mean of three biological replicates.

We previously showed that PFOS and HQ-115 exposure increases the tumorigenicity of TGCT cells *in* vivo (Boyd et al., 2024). However, there was not an increase in *in vitro* cell proliferation (Boyd et al., 2024). To ascertain whether increased tumorigenicity may be due to the effects of PFAS on cell migration, cell migration was assessed in parental 2102EP cells treated with PFOS, HQ-115, and GenX (Fig. 1). The 10 and 1000 nM concentrations used were chosen to represent the mean PFOS human serum levels for general and occupationally exposed populations, respectively, according to the Agency of Toxic Substances and Disease Registry (Agency for Toxic Substances and Disease Registry, 2023). Since consensus-relevant doses of HQ-115 and GenX have not been reported, PFOS concentrations were used. There was no significant difference in the migration rate of cells treated with PFAS (Fig. 1).

### 3.2. HQ-115 alters the metabolomic profile of TGCT cells

**Fig. 2.**
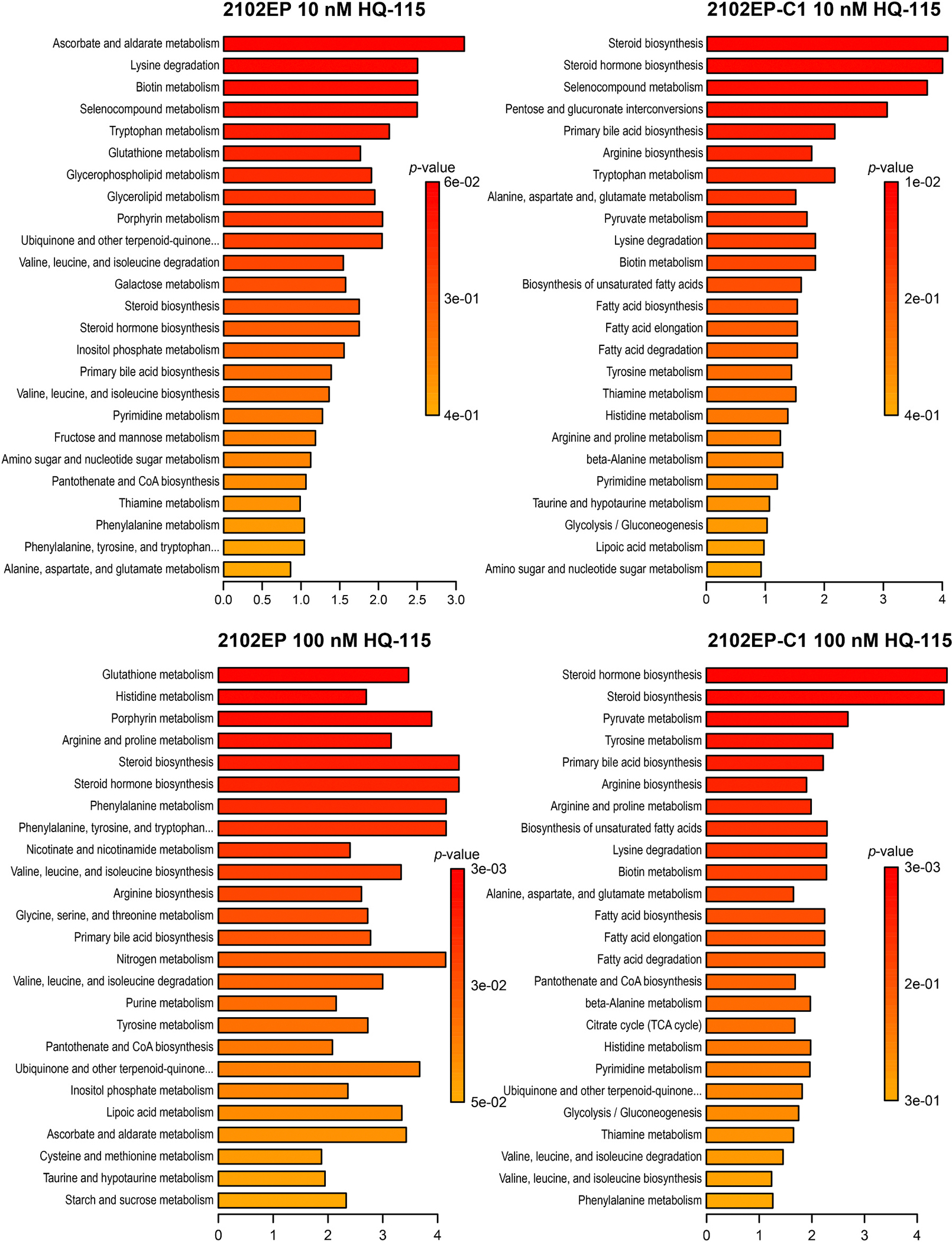
HQ-115 alters metabolite profiles of TGCT cells. GC-MS metabolite profiling of 2102EP and 2102EP-C1 cells treated for 4 days with 10 and 100 nM HQ-115 in biological triplicate. Enrichment Analysis was performed with MetaboAnalyst 6.0.

**Fig. 3.**
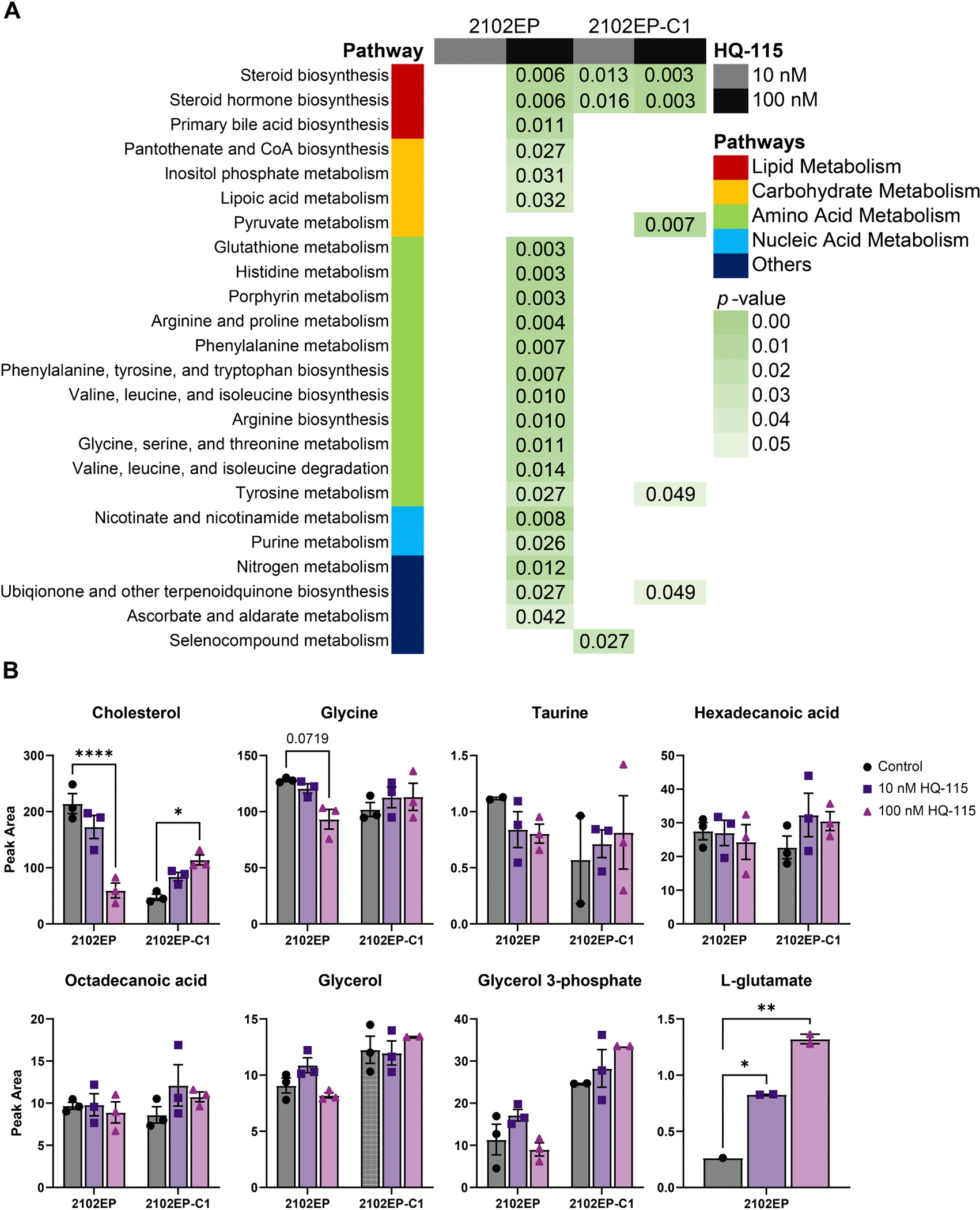
HQ-115 exposure alters metabolite products associated with steroid biosynthesis in TGCT cells. Cells were treated for 4 days with 10 and 100 nM HQ-115 in biological triplicate. (A) Compilation of significantly enriched pathways based on enrichment analysis in MetaboAnalyst 6.0 across cell lines and treatments. (B). Univariate analysis of metabolites involved in lipid metabolism with HQ-115 treatment. Metabolite units are in peak areas adjusted by the internal standard. Data represent mean ± standard error of the mean. * *p* ≤ 0.05, ** *p* ≤ 0.01, **** *p* ≤ 0.0001 compared to control.

In a prior study, we found that PFOS altered the profile of metabolites associated with fatty acid and steroid biosynthesis (Boyd et al., 2024). Because HQ-115 exposure showed a similar increase in tumorigenicity, we were interested in whether HQ-115 had a similar association with changes in lipid metabolism. GC-MS metabolite profiling analysis was performed on 2102EP and 2102EP-C1 cells treated with 10 and 100 nM HQ-115 for 4 days. Principal component analysis (PCA) and hierarchical cluster analysis showed good separation between HQ-115-treated biological replicates from controls (Supplementary Figure S1 and S2). The most prominent metabolic changes were associated with steroid biosynthesis (Figs. 2 and 3 and Supplementary Tables S1). This was surprising since HQ-115 has not been strongly implicated as a lipid mimic compared to the long-chain PFAS, PFOS (Savvidou et al., 2024; Vanden Heuvel et al., 2006).

### 3.3. PFAS exposure alters expression of lipid metabolism genes in TGCT cells

**Fig. 4.**
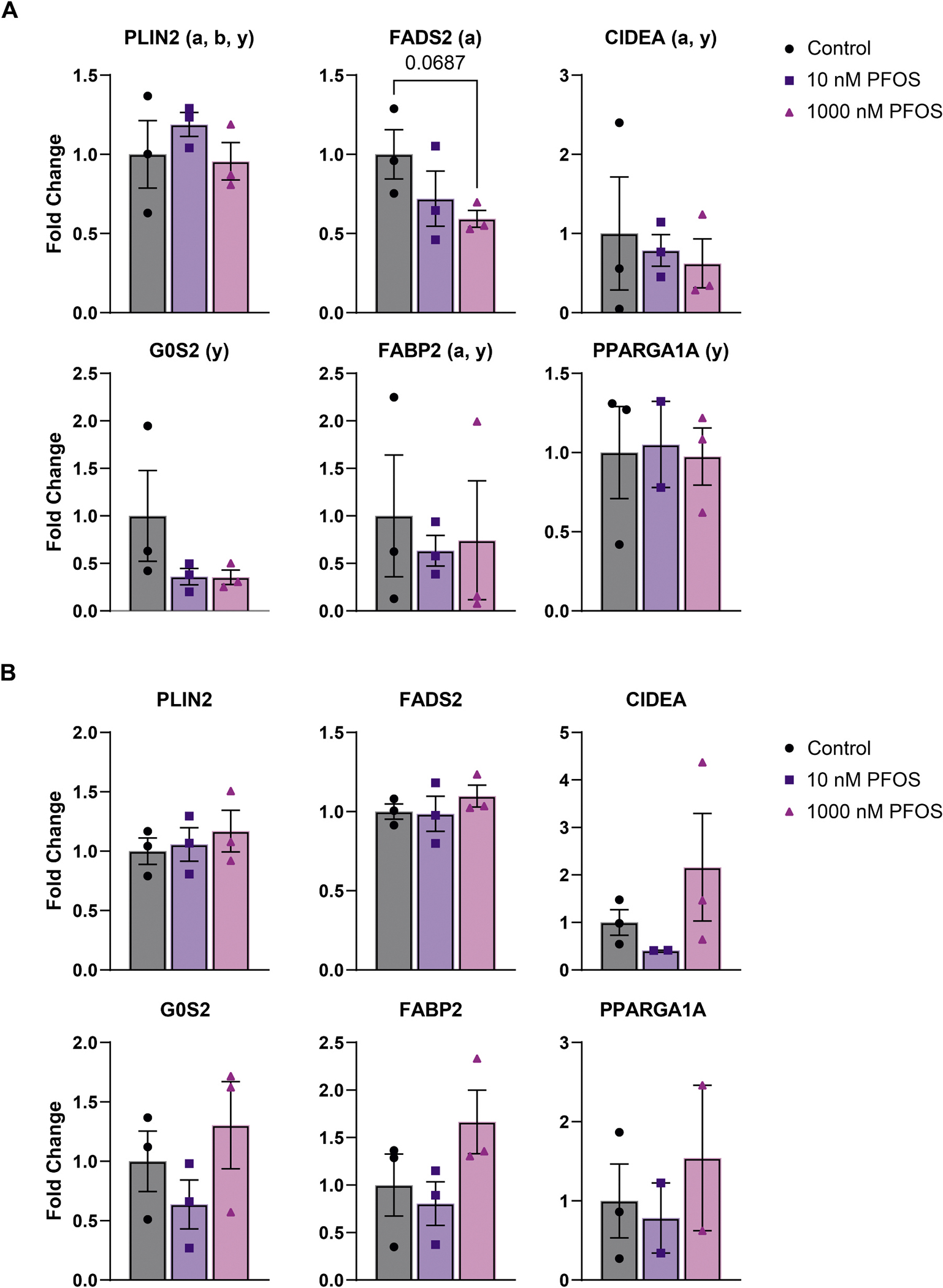
PFOS exposure does not alter transcript levels of select PPAR target genes involved in lipid metabolism. (A) 2102EP and (B) 2102EP-C1 mRNA levels of select genes after 4 days of dosing of the vehicle control, 10 nM PFOS, and 1000 nM PFOS in biological triplicate. Data represent mean ± standard error of the mean. Control, vehicle control; a, PPARα target gene; b, PPARβ target gene; y, PPARγ target gene.

**Fig. 5.**
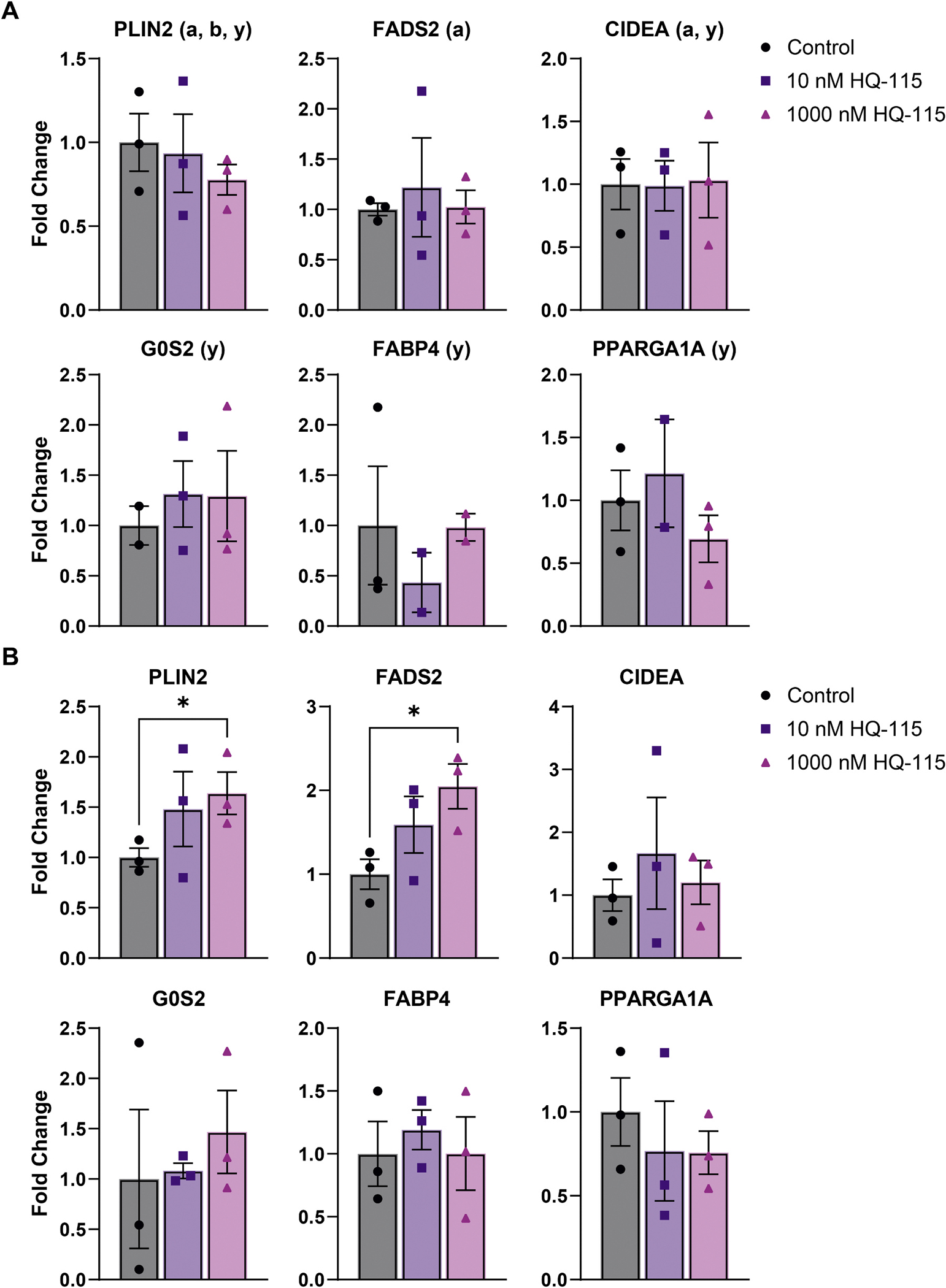
HQ-115 exposure alters transcript levels of select PPAR target genes involved in lipid metabolism in cisplatin-resistant TGCT cells. (A) 2102EP and (B) 2102EP-C1 mRNA levels of select genes after 4 days of dosing of the vehicle control, 10 nM HQ-115, and 1000 nM HQ-115 in biological triplicate. Data represent mean ± standard error of the mean. * *p* ≤ 0.05. Control, vehicle control; a, PPARα target gene; b, PPARβ target gene; y, PPARγ target gene.

**Fig. 6.**
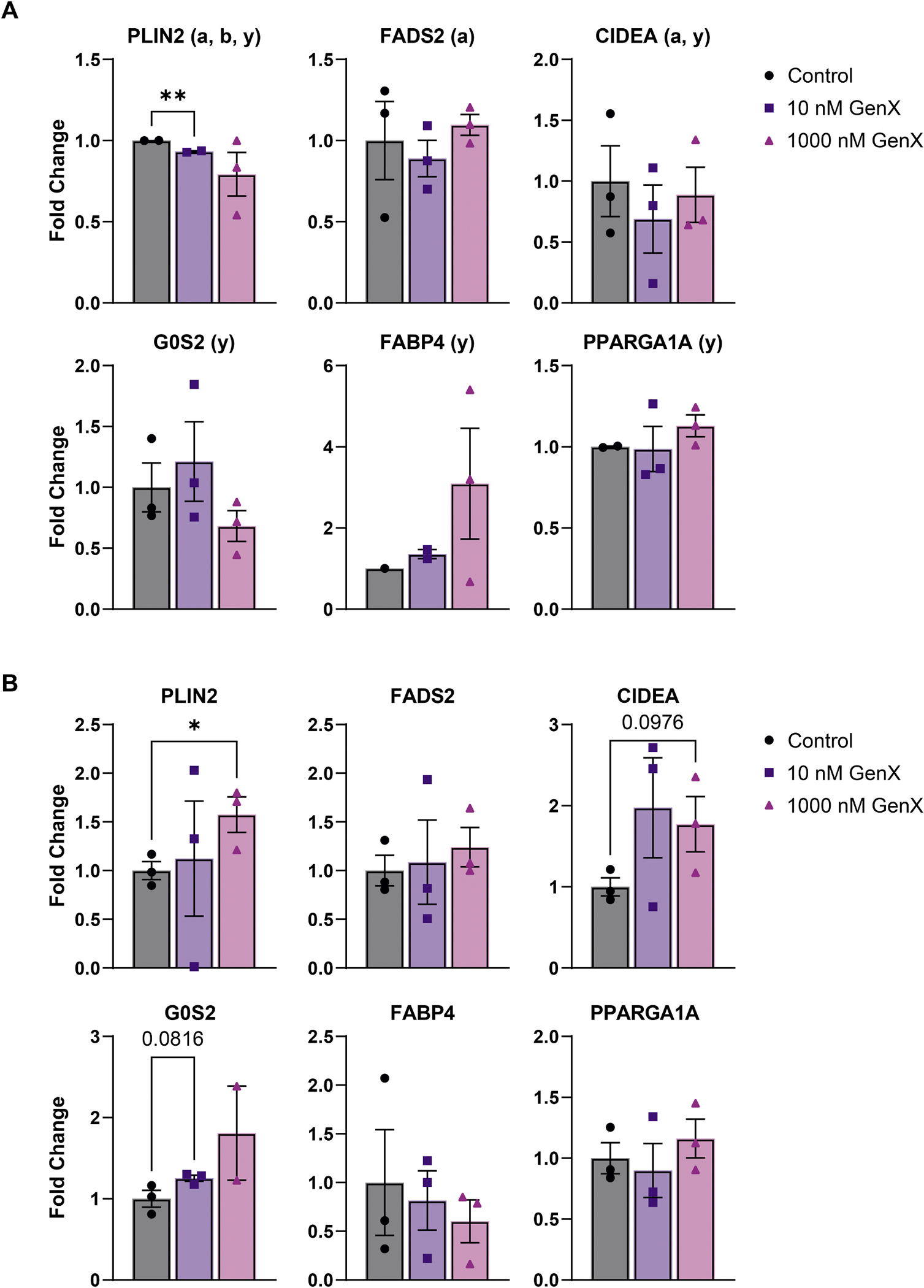
GenX exposure alters transcript levels of select PPAR target genes involved in lipid metabolism in TGCT cells. (A) 2102EP and (B) 2102EP-C1 mRNA levels of select genes after 4 days of dosing of the vehicle control, 10 nM GenX, and 1000 nM GenX in biological triplicate. Data represent mean ± standard error of the mean. * *p* ≤ 0.05, ** *p* ≤ 0.01. Control, vehicle control; a, PPARα target gene; b, PPARβ target gene; y, PPARγ target gene.

Since PPARs regulate the expression of lipid metabolism genes and have been known to bind legacy PFAS, we assessed the ability of PFAS to alter the expression of PPAR target genes in TGCT cells. Genes were selected based on our prior PFOS RNA-seq studies (Boyd et al., 2024), and by consulting experimentally confirmed PPAR target genes in the PPARgene database (Fang et al., 2016). In PFOS-treated cells, there were no changes in the selected PPAR target genes in parental and cisplatin-resistant cells. There was a trend toward decreased *Fads2* expression at the highest dose that warrants further investigation (Fig. 4). For HQ-115-treated cells, there was no change in gene expression in parental cells, but there was an increase in *Plin2* and *Fads2* transcription in cisplatin-resistant cells (Fig. 5). In GenX-treated cells, there was a significant decrease in expression of *Plin2* in parental cells; however, there was a significant increase in *Plin2* expression in cisplatin-resistant cells. There was also a trend for increased expression of *Cidea* and *G0S2* at the highest and lowest doses of GenX, respectively (Fig. 6).

### 3.4. PFAS exposure alters peroxisome-proliferator activation receptor activity in TGCT cells

**Fig. 7.**
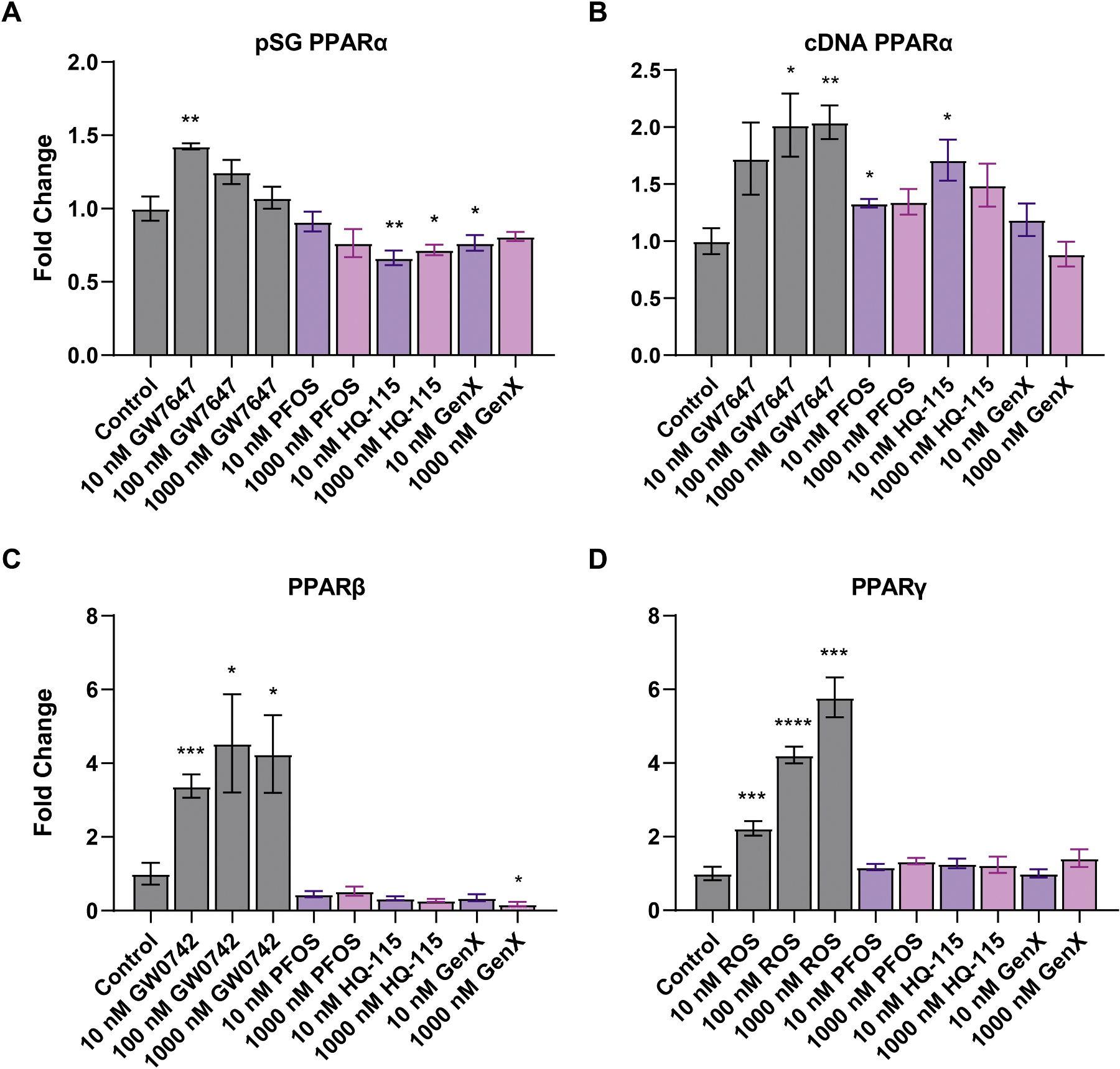
PFAS exposure alters receptor activity of PPARs in TGCT cells. Agonist activity of 10 nM PFOS, 1000 nM PFOS, 10 nM HQ-115, 1000 nM HQ-115, 10 nM GenX, and 1000 nM GenX in 2102EP cells. (A) GW7647, a general PPAR agonist, was used as a positive control for pSG PPARα, a *M. musculus* PPARα. (B) GW7647 was used as a positive control of cDNA PPARα, a *H. sapiens* PPARα. (C) GW0742, a selective PPARβ agonist, was used as a positive control for puro PPARβ, a *M. musculus* PPARβ. (D) Rosiglitazone, a selective PPARγ agonist, was used as a positive control for Sport PPARγ2, a *M. musculus* PPARγ2. Data represent mean ± standard error of the mean of biological triplicates. * *p* ≤ 0.05, ** *p* ≤ 0.01, *** *p* ≤ 0.001, **** *p* ≤ 0.0001. ROS, Rosiglitazone.

To continue the investigation of whether PFAS exposure can affect the activity of PPARs, PPARα, PPARβ, and PPARγ reporter assays were performed. For all receptors, a known PPAR agonist was used as a positive control. PFOS demonstrated partial-agonist activity toward human PPARα at the lowest dose. HQ-115 also showed partial-agonist activity toward human PPARα at the lowest dose. GenX did not show any agonist activity toward any receptor (Fig. 7).

**Fig. 8.**
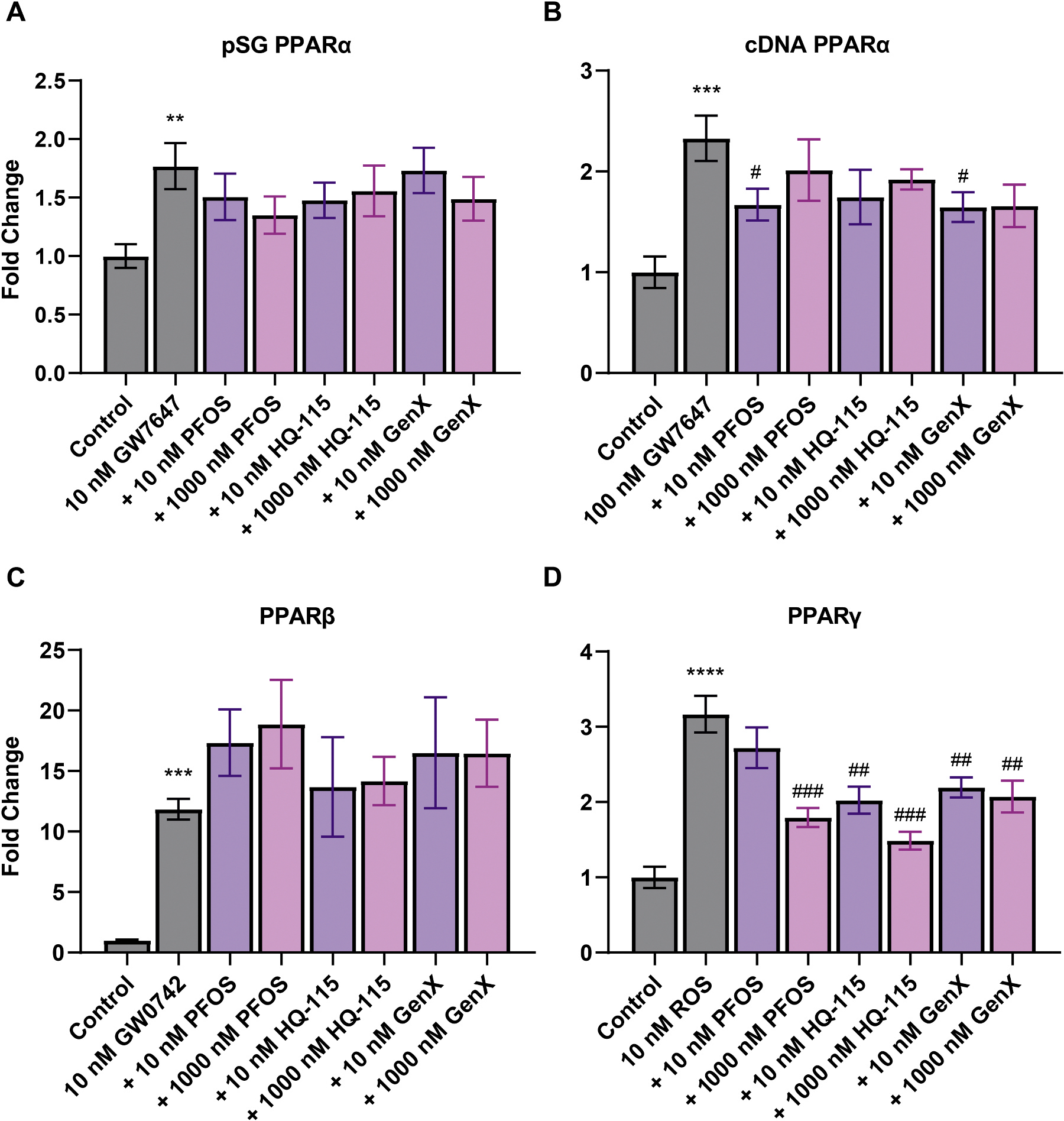
PFAS exposure has antagonist activity toward PPARs in TGCT cells. 10 nM PFOS, 1000 nM PFOS, 10 nM HQ-115, 1000 nM HQ-115, 10 nM GenX, and 1000 nM GenX were dosed in combination with known agonist. (A) 10 nM GW7647 was used as an agonist for pSG PPARα. (B) 100 nM GW7647 was used as an agonist for cDNA PPARα. (C) 10 nM GW0742 was used as an agonist for PPARβ. (D) 10 nM Rosiglitazone was used as an agonist for PPARγ. Data represent mean ± standard error of the mean of biological triplicates. ** *p* ≤ 0.01, *** *p* ≤ 0.001, **** *p* ≤ 0.0001 compared to control. # *p* ≤ 0.05, ## *p* ≤ 0.01, ### *p* ≤ 0.001 compared to positive control. ROS, Rosiglitazone.

Since a subset of the agonist receptor assays suggested partial-activity or antagonist activity, whether PFAS exposure could repress the activity of PPARs in the presence of known PPAR agonists was investigated. PFOS showed antagonist activity toward PPARα at the lowest dose and toward PPARγ at the highest dose. HQ-115 showed antagonist activity toward PPARγ at both doses. GenX showed antagonist activity toward PPARα at the lowest dose and toward PPARγ at both doses (Fig. 8).

## 4. Discussion

Previous studies have implicated lipid metabolism and PPAR activity as mechanisms for PFAS-induced pathogenesis (Boyd et al., 2022; Fenton et al., 2021). This link, however, has not been directly studied in testicular cancer. We previously reported that PFOS and HQ-115 could increase the growth of TGCTs in mice and that PFOS and GenX alter gene expression in TGCT cells that implicate lipid metabolism as a mechanism that warrants further study (Boyd et al., 2024). In this current study, we investigate whether PFAS could affect TGCT cell migration and whether PFAS alters lipid metabolism by disrupting PPAR signaling, both of which may account for the pro-cancer effects of PFAS toward TGCTs. Our study found that PFAS exposure did not alter migration but that HQ-115, like PFOS, alters metabolite profiles associated with lipid metabolism. Further, we provide evidence that PFAS can alter PPAR target gene expression and the activity of PPAR receptors in TGCT cells at doses consistent with human exposure levels.

In our study we compare and contrast PFOS, GenX and HQ115 that are representative of long-chain, short-chain, and clean energy PFAS. The use of a variety of PFAS forms in important since long-chain PFAS are being phased out and replaced by new shorter-chain PFAS forms (Paul et al., 2009; Toxic Substances, 2023; Brase et al., 2021). It will be important to perform similar studies to those described here on newer emerging PFAS including PFAS metabolites and breakdown products from mitigation technologies. In animal studies, exposure to PFOS and HQ-115 increased tumor growth of TGCTs (Boyd et al., 2024). However, there was no increase in cell proliferation *in* vitro (Boyd et al., 2024), prompting us to investigate whether PFAS may have effects on cell migration. Cancer cells utilize migration as a mechanism of increasing tumor size, and PFAS could potentially affect signaling factors regulating cell migration (Friedl and Wolf, 2003). Exposure to PFOS, HQ-115, and GenX did not increase the migration rate in TGCT cells, suggesting that other mechanisms likely account for increased TGCT growth in mice.

The current study found that HQ-115 exposure alters the metabolite profile of TGCT cells, especially alterations linked to lipid metabolism. The results were similar to our previous studies with PFOS (Boyd et al., 2024). PFOS has been previously suggested to act as a fatty acid mimic due to its similarity in structure to fatty acids (Boyd et al., 2022; Vanden Heuvel et al., 2006). Interestingly, HQ-115 has a structure that is quite divergent to fatty acids, yet also altered lipid metabolism, suggesting that PFAS may alter lipid metabolism by additional mechanisms other than fatty acid mimicry. Beyond altered lipid metabolism there were changes suggesting disruption of steroid biosynthesis, mitochondrial function, and membrane lipid remodeling. Alterations in mitochondrial function and lipid membrane homeostasis are known to be involved in cancer promotion (Li et al., 2025; Karthikeyan et al., 2025). Further altered steroidogenesis, especially altered estrogen and androgen levels *in utero*, is a suspected risk factor for testicular cancer development (Bräuner et al., 2021).

A major regulator of lipid metabolism is the nuclear receptor PPARα. PPARα has been previously shown to be involved in PFAS-induced liver toxicity (Li et al., 2019; Das et al., 2017). Expression of perilipin 2 (*Plin2*), a target gene of all three PPARs, was altered by HQ-115 and GenX in TGCT cells. *Plin2* is involved in lipid droplet formation (Cao et al., 2018) and is overexpressed in clear cell renal cell carcinoma (Takahashi et al., 2016). Deletion of this gene increases lipid content and cholesterol in the adrenal glands of mice (Li et al., 2021). An increase in fatty acid desaturase 2 (*Fads2*), a PPARα target gene, was also noted after HQ-115 exposure in 2102EP-C1 cells. FADS is involved in the conversion of polyunsaturated fatty acids into essential fatty acids (He et al., 2018), and expression is altered in several cancers (Chen et al., 2023). Interestingly, FADS2 has previously been associated with poor prognosis and cancer-associated fibroblast infiltration in TGCTs (Chen et al., 2023). However, *Fads2* was actually decreased by PFOS in 2102EP cells, suggesting that effects of PFOS on *Fads* gene expression may be cell context-dependent.

PFAS also had partial agonist activity for human but not mouse PPARα in receptor assays, which is of interest since several studies suggest species-specific differences in the importance of PPARα in PFAS-mediated toxicity (Wolf et al., 2008; Schlezinger et al., 2021; Filgo et al., 2015). Mouse PPARα is reported to be more sensitive to PFAS activation compared to human, although this appears variable, perhaps due to difference in PFAS chain length and other study variables (Wolf et al., 2008; Schlezinger et al., 2021; Filgo et al., 2015). Differences in liver metabolic changes are also seen in humanized PPARα mice compared to wild-type mice (Schlezinger et al., 2021). Our studies differ from other PPAR reporter studies, especially in regard to the widely reported finding that GenX activated PPARα. However, these studies used very high doses of PFAS in the high micromolar range and cells such as liver cells that are known to accumulate high levels of PFAS compared to our studies designed to assess the effects of PFAS at doses achievable in human serum (Evans et al., 2022; Nielsen et al., 2022).

Perhaps the most consistent finding from our studies was that all three PFAS under study demonstrated antagonist activity for PPARγ. There are very few studies investigating the role of PPARγ in PFAS-mediated toxicity, suggesting that in the context of TGCTs, PPARγ may be more sensitive to PFAS exposure. PPARγ has been shown to be upregulated in testicular cancer patients, and a PPARγ inhibitor decreased the proliferation of TGCT cells (Hase et al., 2002). In the normal testis, PPARγ expression is high in Sertoli cells and low in germline cells and plays a role in the regulation of the hypothalamic-pituitary-gonadal (HPG) axis (Olia Bagheri et al., 2021). PPARγ is upregulated by follicle-stimulating hormone and inhibited by luteinizing hormone (Liu et al., 2015). The implication of the HPG axis is especially interesting as the significant increase in endocrine hormones during puberty is implicated in the uncontrolled proliferation of GCNIS cells (Singh et al., 2021; Bräuner et al., 2021). Limitations of our study are the use of two cell lines and the limited number of PFAS forms employed. Future work, including animal and human studies, will strengthen the conclusions obtained here.

## 5. Conclusions

This report investigates the lipid metabolism-altering effects of PFAS on TGCTs as a potential mechanism of tumorigenicity. HQ-115 altered metabolites associated with fatty acid and steroid biosynthesis. *Plin2* and *Fads2* expression was also altered, indicating that lipid formation and utilization have the potential to be affected in PFAS-exposed TGCT cells. Further, the activity of PPARs, known to regulate the expression of genes involved in lipid metabolism, was also affected by PFAS exposure. These results suggest that dysregulation of lipid metabolism may be a potential mechanism for PFAS-mediated pathogenesis of testicular cancer.

## Supplementary Material

MMC1

MMC2

MMC3

MMC4

## Figures and Tables

**Table 1:** Primer sequences for RT-PCR.

Gene	Direction	Sequence
**Fabp4**	Forward	TGGGCCAGGAATTTGACGAA
	Reverse	CACATGTACCAGGACACCCC
**Plin2**	Forward	TGATGGCAGGCGACATCTAC
	Reverse	AAAGGGACCTACCAGCCAGT
**Fads2**	Forward	TTGTGTGTGCGTGTTGTTGG
	Reverse	AGTTCACCAATCAGCAGGGG
**Cidea**	Forward	CTCATCAGGCCCCTGACATT
	Reverse	CCTGTCATGGTTGGAGACCC
**G0S2**	Forward	TGCCACTAAGGTCATTCCCG
	Reverse	ATCAGCTCCTGGACCGTTTC
**Fabp2**	Forward	AACTGAACTCAGGGGGACCT
	Reverse	TGGACTGTGCGCCAAGAATA
**Pparga1a**	Forward	TGAAGGGTACTTTTCTGCCCC
	Reverse	GCACAAACTGGATTCGCCAG
**B-Actin**	Forward	TTTGAGACCTTCAACACCCCAGCC
	Reverse	AATGTCACGCACGATTTCCCGC

## Data Availability

Data will be made available on request.

## Appendix A. Supporting information

Supplementary data associated with this article can be found in the online version at doi:10.1016/j.etap.2025.104866.
