# Supplementary figures and images for: The role of lipid metabolism and peroxisome proliferator activation in mediating pro-cancer phenotypes of poly- and perfluoroalkyl substances in testicular cancer
Source: Environ Toxicol Pharmacol. Author manuscript; Available in PMC 2026 Jul 15. (PMC13371115; doi:10.1016/j.etap.2025.104866)

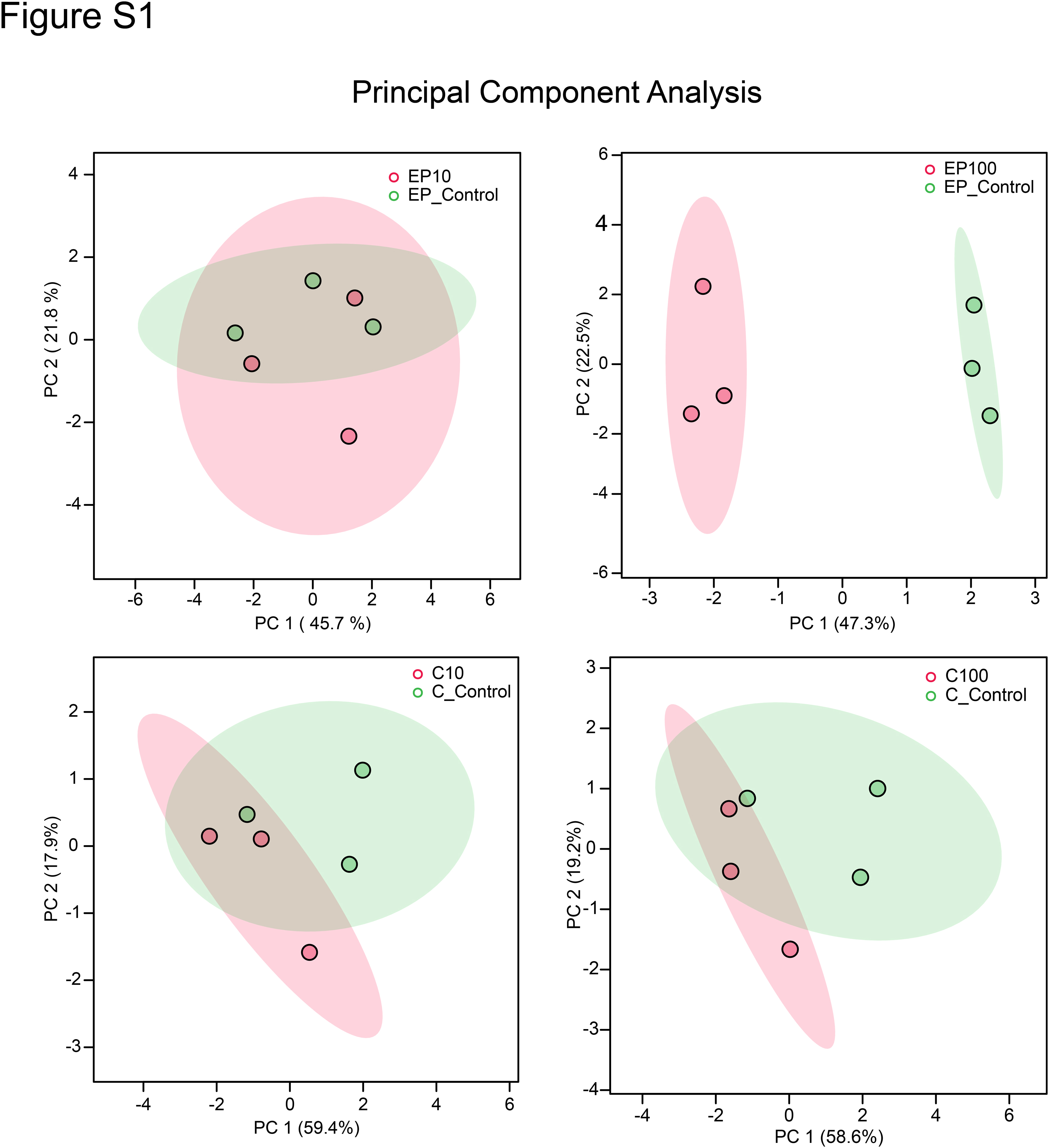

Supplement: MMC3 [file NIHMS2186807-supplement-MMC3.jpg]

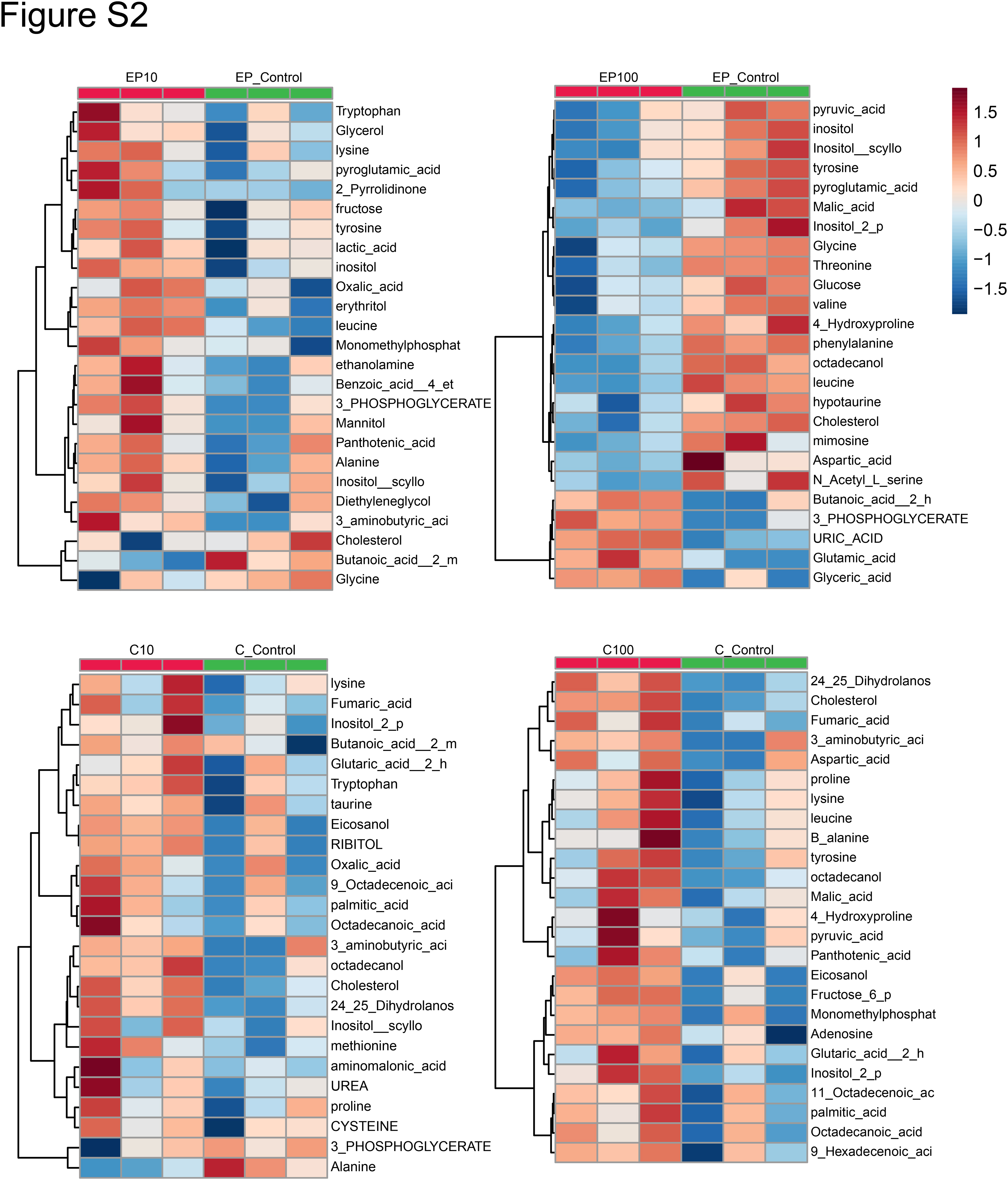

Supplement: MMC4 [file NIHMS2186807-supplement-MMC4.jpg]
